# 3D documentation and classification of incense tree *Aquilaria sinensis* (Lour.) Spreng. wounds by photogrammetry and its potential conservation applications

**DOI:** 10.1002/ece3.11536

**Published:** 2024-06-25

**Authors:** Ho Lam Wang, Tin Hang Wong, Ka Yip Eric Liu, Ho Leung Ryan Tsang, David Tai Wai Lau

**Affiliations:** ^1^ Shiu‐Ying Hu Herbarium, School of Life Sciences The Chinese University of Hong Kong Hong Kong Hong Kong Special Administrative Region China; ^2^ Agriculture, Fisheries and Conservation Department The Government of the Hong Kong Special Administrative Region Hong Kong Hong Kong Special Administrative Region China

**Keywords:** 3D documentation, agarwood, *Aquilaria*, conservation, incense tree, photogrammetry, tree wound classification

## Abstract

In recent years, illegal felling of and damage to the incense tree *Aquilaria sinensis* (Lour.) Spreng. have been reported in Hong Kong. Their native populations are under increasingly severe threat. Therefore, the development of a standard and efficient method to classify and document wounds on vulnerable trees is urgently needed for conservation purposes. In this study, photogrammetry was used to document wounds in *A. sinensis* through 3D modeling. A total of 752 wound records from 484 individual *A. sinensis* trees from Hong Kong were included to establish a new wound classification system. Our major findings include a novel standardized procedure for photogrammetric documentation and a wound classification system. The results of this study will facilitate *A. sinensis* conservation, by enhancing wound documentation and information transfer to law enforcement and education.

## Graphical abstract

The photogrammetric method was adopted to document the wounds of *A. sinensis* in the form of 3D models, and a new wound classification system was established based on 752 wound records from 484 *A. sinensis* individuals in Hong Kong. The study deliverables could facilitate the law enforcement, education, and conservation of *A. sinensis*.

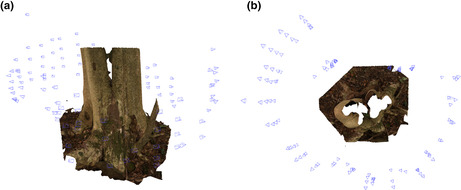

## 1 INTRODUCTION

### 1.1 Background and threatened species assessment of the incense tree (*Aquilaria sinensis* (Lour.) Spreng.)

Trees of the *Aquilaria* genus contain plant tissue that produces a resinous substance commonly known as agarwood, or “Heung” and “Chen‐xiang” (meaning fragrant and sinking incense, respectively, in Chinese) during wound infections. Agarwood is in high demand for incense, traditional medicine, ornaments, and wood‐carved handicraft products (Chua, 2008; Lopez‐Sampson & Page, 2018; Thompson et al., 2022; Yin et al., 2016). To meet the huge demand for agarwood while sustaining the wild population, *Aquilaria* tree cultivation is a common practice in China and Southeast Asia (Ali & Kashem, 2019; Azren et al., 2019; Barden et al., 2000; Chua, 2008; Lee & Mohamed, 2016; Talucder et al., 2016). However, previous studies have shown that the concentrations of oils and aromatic compounds in artificial agarwood differ from those in wild agarwood, which can affect agarwood quality and efficacy (Chen et al., 2017; Espinoza et al., 2014; Ma et al., 2021; Tamuli et al., 2005). One possible explanation is the short agarwood cultivation time due to pressure on farmers from investors to guarantee a constant artificial yield, which limits the time for aromatic compound accumulation in *Aquilaria* trees, whereas wild *Aquilaria* trees may have a longer growing time in which to accumulate more aromatic compounds before harvesting (Devi, 2021; Thompson et al., 2022). Consequently, farmers and investors may be discouraged from participating and investing in artificial agarwood cultivation (Thompson et al., 2022). The current demand for wild agarwood remains high, despite the cultivation of *Aquilaria* trees is widely practiced. Due to overexploitation, wild populations of some *Aquilaria* species, such as *Aquilaria malaccensis* Lam., *Aquilaria rostrata* Ridl. and *Aquilaria crassna* Pierre ex Lecomte, have decreased drastically, and are now classified as ‘Critically Endangered’ on the IUCN Red List of Threatened Species (Harvey‐Brown, 2018a, 2018b, 2018c).

Some *Aquilaria* spp. are native to China and are distributed throughout Hainan, Guangdong, Guanxi, Fujian, Yunnan, and Taiwan (Harvey‐Brown, 2018d; Wang et al., 2007; Xia, 2008). Among the 15 *Aquilaria* species in Southeast Asia, *Aquilaria sinensis* (Lour.) Spreng. (the incense tree, also known as “Tu Chen Xiang”) is the only species found in Hong Kong. Located at the Pearl River Delta, Hong Kong was once an important cultivation site and export port of agarwood from the Tang Dynasty to the Ming Dynasty (619–1644 A.D.), and therefore previously known as “Heung Kong” (meaning fragrant harbor). However, the agarwood industry ceased following pirate attacks in the early Ching Dynasty (1662–1720 A.D.) when incense growers were evacuated from cultivation sites (Hong Kong Herbarium, 2021; Iu, 1983; Lo, 1963). The remaining trees and wild *A. sinensis* gradually developed into rich and healthy local populations, which are commonly found in lowland habitats and mature woodlands throughout Hong Kong (AFCD, 2023; Hong Kong Herbarium, 2021; Iu, 1983; Yip & Lai, 2004). Agarwood obtained from *A. sinensis* is popular for application in traditional Chinese medicine, resulting in uncontrolled exploitation and habitat destruction of *A. sinensis*. Consequently, natural populations in other parts of China have declined by at least 30% in the past 15 years, and the species is now evaluated as ‘Vulnerable’ on the IUCN Red List of Threatened Species (Harvey‐Brown, 2018d; Tian et al., 2009). The few remaining wild populations of *A. sinensis* are threatened by tree destruction and illegal felling (Harvey‐Brown, 2018d; Yin et al., 2016). Hence, Hong Kong may be the last sustained population of wild *A. sinensis* in China (AFCD, 2023; Yip & Lai, 2004).

Since 2005, the *Aquilaria* genus has been scheduled under CITES Appendix II to regulate the trade of their derived materials and products in CITES‐contracted countries. To implement CITES, local law Cap. 586 “Protection of Endangered Species of Animals and Plants Ordinance” regulates the import, export, and re‐export of plants or animals in Hong Kong. In addition to its role in regulating international trade, the local *A. sinensis* populations are also protected from unauthorized vandalism, damage, and felling under Cap. 96 “Forests and Countryside Ordinance” and Cap. 208A “Country Parks and Special Areas Regulations.”

### 1.2 Tree wound documentation

Illegal felling and damage to *A. sinensis* by wound induction have been reported in Hong Kong in recent years (AFCD, 2018; GovHK, 2019; Legislative Council, 2021; Yip & Lai, 2004). Enforcement and prosecution difficulties have further increased owing to scattered poaching sites, advanced tools, and diverse methods for wound induction. Some wound induction methods are so destructive that trees cannot regenerate, causing eventual death or collapse. Hence, continuous poaching over several years threatens the survival of this species in Hong Kong. Previous studies have documented different wound induction methods and technologies for agarwood production from an economic perspective (Akter et al., 2013; Azren et al., 2019; Chowdhury et al., 2016; Talucder et al., 2016). However, a thorough literature review found that there were no official scientific studies documenting wound types in *A. sinensis* from a conservation perspective. Hence, wound documentation and monitoring in *A. sinensis* are essential for obtaining important information, such as the choice of action, wound induction method, wound type classification, and temporal changes in the wounds. All onsite information could provide insights into the establishment of preventive measures and conservation strategies for this vulnerable species in Hong Kong.

Field surveys are a conventional practice in wound documentation of *A. sinensis*, which includes image capture and on‐site wound measurements. However, the complete outline of the trunk and wounds is difficult to represent using two‐dimensional (2D) images because most wounds are three‐dimensional (3D) and irregularly shaped. In addition, wound volume is challenging to measure because only linear parameters, such as the length and width of the wound and the diameter at breast height (DBH) of the trunk, are measured on‐site. Occasionally, additional descriptions of the trunk and wound are included. However, descriptions are usually qualitative and can be subjectively based on the experience and knowledge of field surveyors, which may lead to miscommunication. Some important information, such as the curvature of the trunk and position of the wounds, may not be well recorded by conventional documentation. Furthermore, accurate monitoring of changes in a wound over time, such as the development of a wound infection or agarwood formation, is difficult. Therefore, a standard and efficient documentation method is required to record the three‐dimensional shape and other important parameters of the wounded *A. sinensis*, to improve scientific documentation and knowledge transfer.

### 1.3 New photogrammetry of tree wound

Photogrammetry is the science and technology used to record, measure, and interpret objects in photographic images (Awange & Kyalo Kiema, 2013; Schenk, 2005). It includes a major technique called “Structure from motion (SfM)” which estimates 3D structures of objects from 2D image sequences by finding the correspondence between images (Eltner & Sofia, 2020; Schonberger & Frahm, 2016). Over the past decade, photogrammetry has been used in many botanical studies to document the 3D structure of plants. In general, the 3D structures of plants can be reconstructed by overlapping adjacent images captured from different angles. Common photogrammetric outputs of plant documentation include point clouds and 3D meshes, both of which provide 3D views of plant structures. A point cloud is a collection of points in a 3D coordinate system representing the surface of an object. Point clouds can be classified as sparse or dense based on the density and number of points, respectively. A sparse point cloud is usually generated first during reconstruction to show the preliminary structure of 3D models and serves the purpose of matching overlapped images. With sufficient quality and overlapping images, a dense point cloud can be reconstructed to show the fine structures of 3D models. Point clouds can be further reconstructed into a mesh, in which the points are connected to form polygons that represent object surfaces. Point clouds and 3D meshes have aided scholars and botanists in studying plant morphology, structure, and growth. Photogrammetry has been used to determine tree structures, from which the results demonstrated high accuracy in measuring tree height, stem diameter, and stem surface structure (Mokroš et al., 2018; Morgenroth & Gómez, 2014; Surový et al., 2016). Tree diameter measurements derived from the photogrammetric method are highly correlated with destructive diameter measurements in irregularly shaped tree trunks (Bauwens et al., 2017). In addition, photogrammetry has been used to monitor annual trunk increments, resulting in <1% error compared to tape measurements (Mokroš et al., 2020). Although laser scanning (also known as Light Detection and Ranging) can accurately reconstruct 3D structures for tree measurements (Cao et al., 2021; Ferraz et al., 2016; Kwak et al., 2007), one notable advantage of photogrammetry in this study is that the photogrammetric 3D model reflects the genuine color of the objects. Color is an important morphological characteristic for plant authentication; therefore, photogrammetry can be a helpful tool for reconstructing plant 3D models for scientific purposes. Recently, photogrammetric techniques have been used to reconstruct textured carpological specimens (Wang et al., 2022) and flowers (Leménager et al., 2022). The resulting reconstructions illustrate plant morphology, which is potentially useful in plant taxonomy and morphological studies. The cost of 3D photogrammetry methods for reconstructing 3D models for plant documentation is lower than that of other methods (Andújar et al., 2018; Dong et al., 2021). In addition, 3D photogrammetry is nondestructive during plant observation and measurement (Arief et al., 2021; Bauwens et al., 2017; Mokroš et al., 2020; Tirrell et al., 2023). Therefore, 3D photogrammetry has great potential for use in tree wound 3D documentation. However, no studies have been conducted on the application of photogrammetric methods to document tree wounds, specifically in *A. sinensis*.

This study provides a complete documentation of wound types found on the vulnerable tree species *A. sinensis*, with detailed classifications and descriptions of all wound types discovered in Hong Kong over the last 6 years. In addition, this study demonstrates the practical application of the photogrammetric method to wound documentation in *A. sinensis*. All wound types were well represented in line drawings and 3D photogrammetric models. Hence, the results of this study provide a standardized method for 3D wound documentation and determination with the aim of facilitating *A. sinensis*. conservation worldwide.

## 2 METHODS

### 2.1 Study area

According to the Agriculture, Fisheries, and Conservation Department (AFCD), the Government of the Hong Kong Special Administrative Region which conducts regular *A. sinensis*. surveys and wound monitoring in Hong Kong, *A. sinensis* are generally found in lowland habitats throughout Hong Kong, particularly in mature secondary forests.

All *A. sinensis* inspected in this study were recorded in lowland habitats. Among these sites, five illegal poaching hotspots were selected for 3D wound documentation to showcase wound type diversity.

### 2.2 Wound classification

Wound assessment and classification were conducted for a total of 484 mature *A. sinensis* trees. Trees with a trunk diameter of ≥95 mm at a height of 1.3 m above ground level were defined as mature trees, based on the relevant guidelines issued by the local government (AFCD, 2006; DEVB, 2020). All individuals were first recorded and examined for wounds in the field. Close‐up photographs were taken upon wound identification for further classification of wound type in the laboratory. The classification criteria included (i) wound shape, (ii) wound position, (iii) wound dimensions, (iv) wound induction method, and (v) agarwood maturity. Wound‐type designations were based on the shape, position, and induction methods. In addition, a vertical level was set at breast height (1.3 m above ground level) to differentiate the upper and lower parts of the trunk when identifying saw‐induced wounds, as sawing and axing seem to be more frequently applied at or below breast height.

### 2.3 Wound selection for 3D documentation

For each wound type, one to two *A. sinensis* individuals were selected for 3D documentation. The selected individuals had typical and representative wounds within the assigned wound category. Individuals with multiple wounds were more suitable for 3D documentation, as documenting all wound types was more time‐effective and representative of the tree wound extent of *A. sinensis* populations in Hong Kong. In this study, *A. sinensis* individuals in inaccessible areas, such as steep slopes, cliffs, and rocky terrain, were not included in the imaging process for safety reasons. In addition, setting up lighting devices and capturing wounds from all angles is difficult and risky due to the topography of these inaccessible areas.

### 2.4 3D documentation of *A. sinensis* wounds

The 3D documentation method used in this study was derived from a previous study (Wang et al., 2022). Modifications were made to the tools and image acquisition procedures, and the new method was repeatedly validated. The 3D documentation procedures of the wounds were well‐established and documented as follows.

#### 2.4.1 Tools and devices for image acquisition

To capture images, a mirrorless Canon EOS R5 camera was fitted with a Canon EF 35 mm f/2 IS USM prime lens. The prime lens was used, as its focal length is suitable for capturing images of large objects (e.g., tree trunks) and produces better results than that of a zoom lens in 3D model reconstruction. As light was shed from the understory layer of the forest, four Phottix M200R RGB Light plates were used to provide sufficient lighting. The light plates on the tripods were placed in four directions from the main trunk with a rotation of ~90° towards each other to ensure uniform lighting. A ruler was placed on the trunk as a measurement marker to determine the true wound dimensions. Camera configurations and other tools are listed in Table 1.

**TABLE 1:** Configuration of camera and other tools.

Camera	Canon EOS R5
Lens	Canon EF 35 mm f/2 IS USM
Image resolution	4K (4176 × 2784 pixels) 8K (8192 × 5464 pixels)
Shutter Speed	1/200 s
Aperture	f/8
ISO	3200 to 4000
Color temperature of camera	5600 K
Color temperature of light plates	5600 K
Light intensity of light plates	100%

#### 2.4.2 Image acquisition

Handheld shooting was used to capture close‐up images of trunks and wounds. The camera was located one to two meters away from the tree. The camera was positioned in such a way that it directly faced the trunks without any upward or downward tilting. Images were captured from top to bottom while circling the trees at 15°–25° intervals between each shooting point. When capturing structures >2 m high, the camera angle was tilted upwards. In some cases, additional photos of complicated wounds with irregular shapes and structures were taken.

#### 2.4.3 3D model reconstruction

The images were analyzed in a photogrammetric software 3DF Zephyr (version 7.005; https://www.3dflow.net/) for image matching and 3D model reconstruction under a high‐ranked computing system (CPU: Alienware Aurora R15 with a 13th Gen Intel® Core™ i9 13900KF; GPU: NVIDIA GeForce RTX 4090). The reconstruction process was identical to our previous methodology (Wang et al., 2022), with one alteration: a decimation filter was applied to the mesh after “Surface Reconstruction” to minimize the file size for efficient storage and display. The number of target vertices was adjusted to 350,000.

#### 2.4.4 3D model editing

First, the 3D model was imported into the editing software Blender (version 3.4; https://www.blender.org/) for manual editing. Second, the 3D model orientation was fixed and the background structures were removed. Third, the 3D model was scaled to the actual size using a ruler on the trunk. Last, the final 3D model and texture were exported as wavefront files (.obj), and texture (.jpeg or .png) respectively.

## 3 RESULTS

### 3.1 Reconstructed 3D models of *A. sinensis* wounds

Eighteen 3D models of *A. sinensis* wounded trunks were reconstructed. The average time for image acquisition was ~30 mins, depending on the complexity and size of the wound. An ID number was assigned to each individual tree. Information regarding the reconstructed 3D models is presented in Table 2.

**TABLE 2:** Information of the 3D models of *A. sinensis*.

Tree code	No. of photos captured	No. of photos matched	Resolution	Mesh size (MB)	Texture size (MB)	Reconstruction time (min)
QH564	319	319	8K	78.4	60.0	70
QH568	416	416	8K	75.6	70.9	116
QH569	305	305	8K	78.3	35.2	56
QH573	292	267	8K	77.5	50.9	55
STK074	331	331	8K	81.2	58.2	76
STK075	333	333	8K	76.5	42.2	60
T106	165	165	4K	75.0	16.7	11
T107	122	122	4K	74.7	19.6	8
TH_T11	178	178	4K	76.4	21.8	12
TH028	160	160	4K	66.5	9.45	9
TH038	112	112	4K	75.0	21.7	11
TH118	177	177	4K	76.7	19.2	15
TH147	147	147	4K	73.5	10.5	10
TH243	143	143	4K	74.3	14.6	13
TL039	547	542	8K	76.2	51.3	120
TL049	196	196	8K	76.3	24.4	35
TL097	329	329	8K	76.0	30.7	60
TY061	197	197	4K	75.1	27.8	16

**FIGURE 1:**
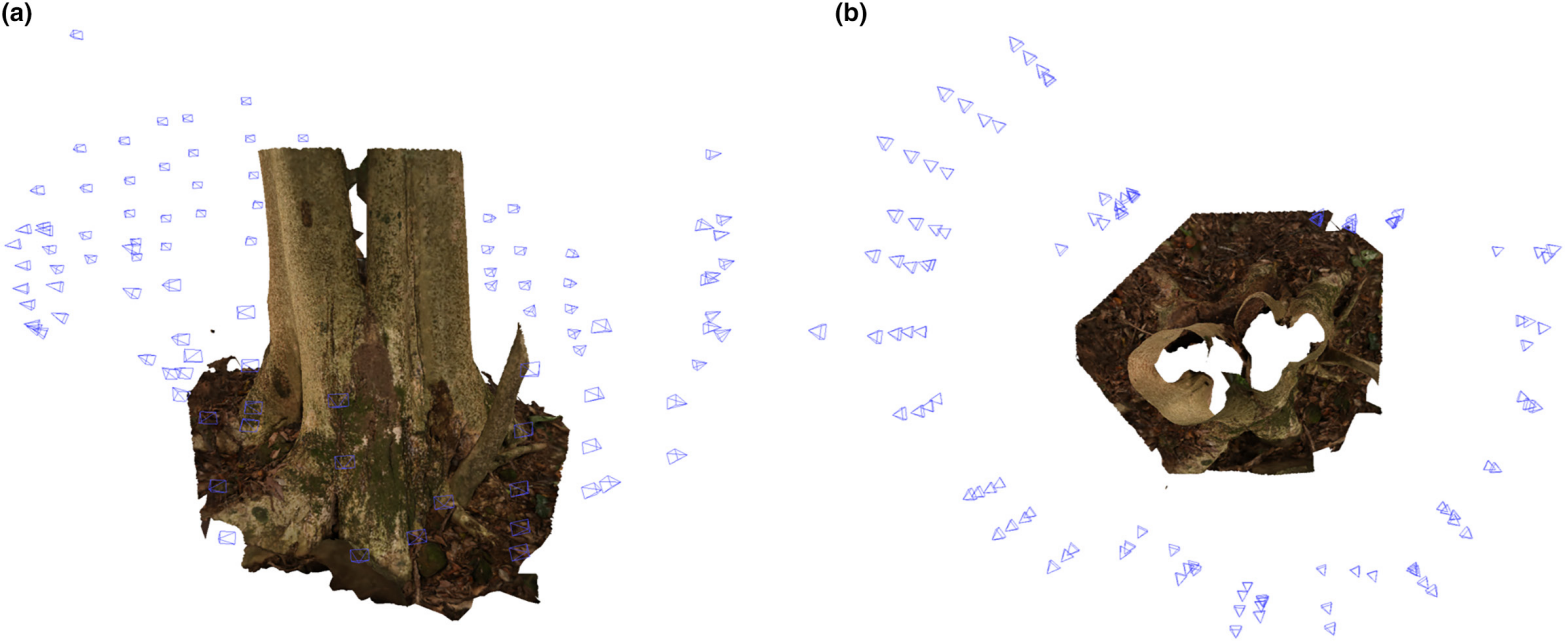
The shooting angles of the camera. A reconstructed 3D model of TH038. The camera orientation was estimated by the photogrammetric software 3D Zephyr and shown as a pyramid. (a) The camera orientation from side view. (b) The camera orientation from top view.

All the 3D models were uploaded to the “Virtual Carpological Herbarium” in the Shiu‐Ying Hu Herbarium Archive System (https://syhuherbarium.sls.cuhk.edu.hk/collections/3d‐digitized‐tag/wound/; Username: syhuherbarium; Password: @CUHK) under a new category of “Incense Tree wound classification system.” To browse the 3D models with ease, the following hardware and software configurations are recommended: for desktop or laptop computers, CPU: Intel i5 2.1G or above; RAM: 6G or above; 3D GPU: CUDA‐enabled graphics card (e.g., Nvidia GeForce GTX 1650 or above); web browsers: Chrome 93.0.4577.82 or above/ Firefox v92.0/ Edge v93.0.961.47/ Safari v5.1.7/above. For mobile or tablet, OS: Android 9 or above/ iOS 14.8 or above (Figure 1).

### 3.2 Novel wound classification system of *A. sinensis*

**FIGURE 2:**
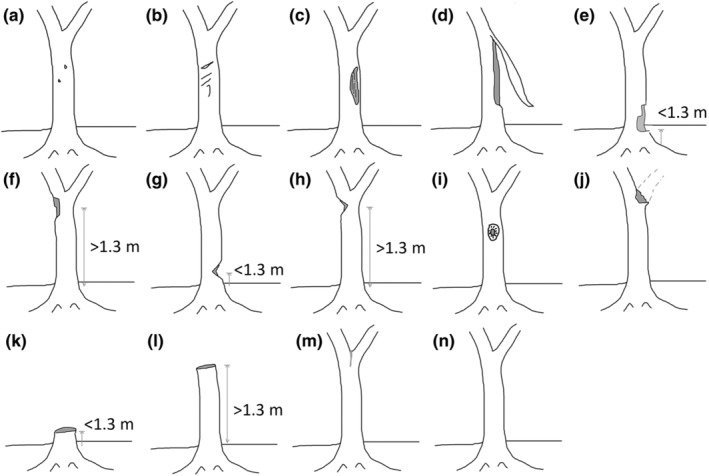
Illustration of the wound types on *A. sinensis*. (a) Drilled‐hole patches. (b) Slash patches. (c) Peeled bark. (d) Vertical cleaving. (e) Horizontal sawed below breast height. (f) Horizontal sawed above breast height. (g) Angle sawed below breast height. (h) Angle sawed above breast height. (i) Chiseled. (j) Side branch removal. (k) Trunk removal below breast height. (l) Trunk removal above breast height. (m) Natural wound between branches. (n) A non‐damage trunk.

**FIGURE 3:**
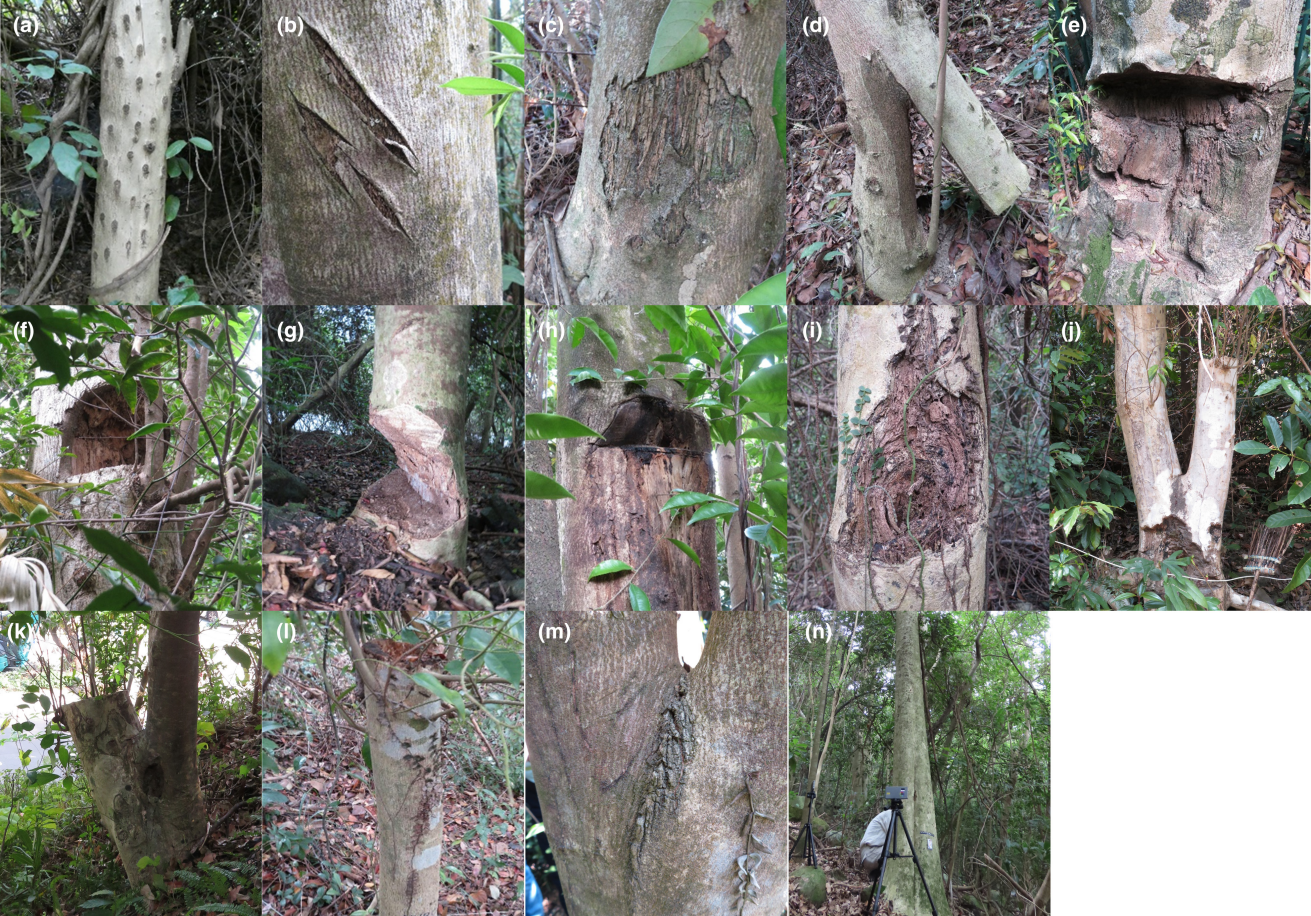
Reference photos of the wound types of *A. sinensis*. (a) Drilled‐hole patches. (b) Slash patches. (c) Peeled bark. (d) Vertical cleaving. (e) Horizontal sawed below breast height. (f) Horizontal sawed above breast height. (g) Angle sawed below breast height. (h) Angle sawed above breast height. (i) Chiseled. (j) Side branch removal. (k) Trunk removal below breast height. (l) Trunk removal above breast height. (m) Natural wound between branches. (n) A non‐damage trunk.

A total of 802 on‐site records, including 752 artificial wounds, 14 natural wounds, and 36 non‐damaged trunks, from 484 *A. sinensis* were recorded in Hong Kong. On the basis of these observations, we developed a new wound classification system. Thirteen wound types were classified, including 12 artificial and one natural wound type. An additional undamaged trunk was used as a reference. All wound types were named according to their morphology and the associated wound induction methods. All wound types were well illustrated by line drawings (Figure 2) and typical representative photos of each type (Figure 3). The wound types were classified into three categories according to the purpose of induction: wound induction for (i) agarwood formation, (ii) agarwood collection, and (iii) both agarwood formation and collection (Table 3). The characteristics of each wound type are described below.

- *Drilled‐hole patches* (鑽孔): Small circular hole patches were induced on the tree trunk. The hole diameter was <50 mm. This type of wound was induced during agarwood formation. Wound induction methods included drilling, burning‐chisel drilling, and injection of agarwood inducers or fungi (Azren et al., 2019; Chowdhury et al., 2016; Talucder et al., 2016).
- *Slash patches* (割痕): Long scars with <20 mm depth were induced on the tree bark. This type of wound was induced during agarwood formation. Methods of wound induction included axing, knife chopping, and partial trunk pruning (Chowdhury et al., 2016; Talucder et al., 2016).
- *Peeled bark* (剝皮): The tree bark was removed, and the cambium layer was exposed. This type of wound was induced during agarwood formation. Wound induction involved bark peeling (Akter et al., 2013; Chowdhury et al., 2016).
- *Vertical cleaving* (縱向分裂): Part of the trunk was cleft and tilted at an angle from the remaining part of the trunk. The tree remained alive in this situation. This type of wound was induced during agarwood formation. The method of wound induction involved cleaving the trunk vertically using an axis or chopper, and obstacles, such as stones, which might be placed at the cleaving point to tilt the trunk.
- *Horizontal sawed below breast height* (胸下橫鋸): Part of the trunk was removed with the horizontal cutting edge and the wound was located at <1.3 m from the ground. This type of wound was used for agarwood formation and collection.
- *Horizontal sawed above breast height* (胸高橫鋸): Similar to the characteristics described in “(e) Horizontal sawed below breast height,” but the wound was located at >1.3 m from the ground.
- *Angle sawed below breast height* (胸下斜鋸): Part of the trunk was removed with an angled cutting edge and the wound was located at <1.3 m from the ground. This type of wound was used for agarwood formation and collection.
- *Angle sawed above breast height* (胸高斜鋸): Similar to the characteristics described in “(g) Angle sawed below breast height,” but the wound was located at >1.3 m from the ground.
- *Chiseled* (鑿孔): An irregularly‐shaped hole was found on the trunk. The chiseled holes recorded in this study were usually found above breast height. The diameter of the hole was >50 mm. This wound type was induced for agarwood formation and collection by using the hole digging method.
- *Side branch removal* (分枝鋸斷): The side branch of the tree was completely chopped. This type of wound was induced for agarwood collection using a sawing method.
- *Trunk removal below breast height* (胸下鋸斷): The tree trunk was felled so that only the stump remained. The removed trunk was further exploited by poachers to find the agarwood embedded inside. The cut was located <1.3 m from the ground. This type of wound was induced for agarwood collection using a sawing method.
- *Trunk removal above breast height* (胸高鋸斷): Similar to the characteristics described in “(k) Trunk removal below breast height,” but the cut was found at >1.3 m from the ground.
- *Natural wound between branches* (枝間自然裂傷): Wounds were induced naturally between two diverging branches.

**TABLE 3:** Purpose and the percentage of occurrence of artificial wound types of *A. sinensis*.

Wound type	For agarwood formation	For agarwood collection	No. of wounds	Percentage
Drilled‐hole patches	Yes	No	7	0.9
Slash patches	Yes	No	44	5.9
Peeled bark	Yes	No	163	21.7
Vertical cleaving	Yes	No	9	1.2
Horizontal sawed below breast height	Yes	Yes	65	8.6
Horizontal sawed above breast height	Yes	Yes	19	2.5
Angle sawed below breast height	Yes	Yes	92	12.2
Angle sawed above breast height	Yes	Yes	37	4.9
Chiseled	Yes	Yes	47	6.3
Side branch removal	Yes/No	Yes	102	13.6
Trunk removal below breast height	Yes/No	Yes	35	4.7
Trunk removal above breast height	Yes/No	Yes	132	17.6
Total	N/A	N/A	752	100

### 3.3 Wound occurrences of *A. sinensis* in Hong Kong

Percentage occurrence of all artificial wound types in *A. sinensis* are given in Table 3. Observations of natural wounds and undamaged trunks are excluded from the percentage calculation. Among the 12 types of artificial wounds, the top three are “Peeled Bark” (21.7%), “Trunk Removal above breast height” (17.6%) and “Side Branch Removal” (13.6%). The least recorded wound type is “Drilled‐hole Patches” (0.9%). The total number of sawed wounds induced below breast height (i.e. “Horizontal Sawed below breast height” & “Angle Sawed below breast height”) is almost three times higher than those induced above breast height (i.e. “Horizontal Sawed above breast height” & “Angle Sawed above breast height”). The former accounts for 157 wounds, and the latter for 56. Conversely, the number of “Trunk Removal below breast height” is about one fourth of “Trunk Removal above breast height”, in which the former accounted for 35 wounds while the latter accounted for 132 wounds.

## 4 DISCUSSION

### 4.1 Advantages of the photogrammetric method and 3D models resulted from this study

This study demonstrated photogrammetry application for 3D documentation of *A. sinensis* wounds. Eighteen 3D models were constructed, each representing a unique *A. sinensis* individual tree occurring in Hong Kong. The 3D modeling used in this study successfully documented the complete morphology of tree trunks with wounds, in which the structures could be clearly observed from all angles. As the color temperature of the light source was adjusted to 5600 K, the 3D models reflected the genuine color of the trunk and wounds, which is a prerequisite for distinguishing between individuals and the corresponding wound development stages.

Compared with the conventional documentation of wounds in the form of 2D photos and measurements, the new method derived from this study reconstructs the complete structure of wounds using 3D models. Therefore, we demonstrate that the 3D models can facilitate better wound documentation for research, and further enhance information transfer from field surveyors to researchers or other users.

The average size of the 3D model, including the mesh (.obj) and texture (.jpg) was ~100 MB, which is equivalent to 16–17 images at 4K resolution. In the early stages of this study, the 3D models were reconstructed and exported without applying a decimation filter. The file size was too large (>2 GB) for certain 3D models. A large file is difficult to store and display. Hence, a decimation filter was applied after “Surface Reconstruction” to reduce the number of vertices in the mesh, which significantly reduced the file size. Furthermore, the small file size increased the storage efficiency and reduced the processing time when displaying 3D models in an online database. Furthermore, the 3D image quality remained high and unchanged, even keeping a large number of 3D images in the database. The manual instruction for the decimation filter application can be found in the software or on the 3D Zephyr website (http://3dflow.net/zephyr‐doc/3DF%20Zephyr%20Manual%206.0%20English.pdf).

### 4.2 Preparation time and post‐processing work

Although the average processing time of a 3D model from image acquisition to reconstruction is ~72 mins, more time is invested in preparation and post‐processing. The AFCD team has conducted numerous field surveys over the past few years to gather detailed information of *A. sinensis* in Hong Kong, consolidating the wound classification system used in this study. A pre‐trip survey was conducted by the AFCD team before each 3D wound documentation to ensure the suitability of the wound status and condition. A tremendous amount of time and manpower was involved in the preparation. Moreover, post‐processing tasks such as 3D model editing, data input, and the management of online databases, servers, and webpages are complicated and resource‐demanding. Hence, the average processing time of the 3D model in this study was only a reference based on the core technical procedures. Time and resources required for preparation and postprocessing should be considered when implementing similar research studies and databases.

### 4.3 Challenges of 3D documentation by the photogrammetric method

A few challenges were encountered during image acquisition in this study. One major challenge was the dark environment of forest understory layers, as most of the sunlight in the canopy layer was blocked. Therefore, inadequate sunlight was available for photography. Although light plates were used as lighting, the sensitivity of the camera had to be adjusted to ISO 3200‐4000 to adapt to good imaging under such dark environments, and some image noise was inevitable. Generally, a stronger light source was required to reduce the image noise. However, occasional intense sunlight resulted in a high contrast in the same image. Some structures were overexposed, whereas others were underexposed. In such cases, the software was not able to recognize these structures for further 3D model reconstruction. Even when recognized by the software, the color was too bright or too dark, and therefore the image was not a genuine representation. This problem was usually found in trees at the forest edge, where one side of the tree faced an open area with direct sunlight, while the other side faced the inner forest with weak sunlight. Light contrast from strong sunlight still existed even when the light plates were used. To minimize light contrast, image acquisition in future studies should be conducted early in the morning or evening to avoid intense sunlight. Meanwhile, the variation in sunlight can create unstable light conditions, which may impair the image‐matching procedure because it is difficult for the software to recognize tie points from overexposed or underexposed images. To date, this problem has only been found in one batch of images from QH573, in which 25 of the 292 images were not recognized by the software. In most cases, the image acquisition of an individual tree should be completed within 30 mins to 1 h to minimize the problems caused by sunlight variations.

In addition, excessive information from the background environment, such as the landscape and surrounding vegetation, can reduce 3D model reconstruction accuracy. Many *A. sinensis* individuals documented at the study sites were surrounded by shrubs and lianas, and some grew close to other trees. These surrounding plants covered part of the trunk and wounds or even blocked the image shooting angles. Unfortunately, some images of the trunk and wounds were not properly captured. Consequently, the image‐matching procedure was impaired because the software had difficulty in recognizing the tie points from these images. Moreover, irrelevant information from the background environment can increase image processing time, which reduces the efficiency of 3D model reconstruction. In this study, the file size of the 3D model increased when the unwanted surrounding vegetation was also created as a mesh, which occupied more storage capacity. To address this problem, the obstacles around the trunk and wounds were shifted for better accessibility and image acquisition from all angles. However, small trees and large shrubs that could not be shifted were reconstructed as part of the final 3D model. The reconstructed excess mesh was deleted after the “Surface Reconstruction” to minimize the file size. A trial was conducted on TY061 to compare file sizes before and after removing excessive mesh. The original mesh size and texture were 404 and 628 MB, respectively, and were reduced to 186 and 294 MB, respectively, after the removal of the excess mesh. With the application of the decimation filter, the mesh size and texture size were further reduced to 75.1 and 27.8 MB, respectively. Hence, removing irrelevant background information is critical for maintaining an essential file size for efficient storage and experimentation.

### 4.4 Advantages of the novel wound classification system of *A. sinensis*

The university research and government conservation teams collaborated to validate the raw data and whole procedures throughout the study, hence a novel wound classification system for *A. sinensis* was established. A wound classification system is critical for monitoring *A. sinensis* in Hong Kong as it provides a standardized method for classifying wound types. Together with the 3D wound documentation method used in this study, our novel wound classification system can facilitate wound determination by field surveyors and prevent subjective observations that could lead to miscommunication. Hence, it enhances information transfer from field surveyors to researchers to understand the exact wounding situation. In addition, detailed descriptions of the associated wound induction methods are provided in the classification system, which can facilitate some of the current conservation actions, such as tree guard installation, patrol planning, and application of wound dressing oil (AFCD, 2018).

### 4.5 Analysis of the wound diversity of *A. sinensis*

Our results showed that a wide variety of *A. sinensis* wound types were recorded in Hong Kong, including 12 artificial wound types. Some individuals had more than one type of wound, thus suggesting that poachers used various methods and tools to induce wounds in *A. sinensis* possibly for enhancing agarwood yield.

Among all wound types, “Peeled Bark” was the most frequent in Hong Kong. This type of wound is usually induced by removing tree bark through peeling and tearing so that the cambium layer is exposed to air and causes agarwood formation. “Peeled bark” is likely the most frequent wound type in Hong Kong due to the simple wound induction method that requires only a knife or an axis and little effort, whereas other induction methods are more complex. Indigenous people in Bangladesh and other regions peel the bark to promote wound infection and seasonally harvest wood chips from live trees (Akter et al., 2013; Chowdhury et al., 2016). Therefore, *A. sinensis* individuals in Hong Kong with “Peeled bark” are likely to be revisited by poachers, and this wound type could potentially be used as an indicator to track down poachers.

“Drilled hole patches” were the least frequent wound type of *A. sinensis* in Hong Kong. One individual, TY061, was found to have up to 90 drilled holes on its trunk, which were evenly distributed. Therefore, this individual will be revisited regularly to monitor wound changes in the future study. Such wounds may be induced by drilling or burning‐chisel drilling, possibly followed by injecting an agarwood inducer or fungi into the holes to accelerate agarwood formation (Akter et al., 2013; Azren et al., 2019; Chowdhury et al., 2016; Talucder et al., 2016). Therefore, *A. sinensis* individuals with “Drilled‐hole patches” will be revisited for further wound records and potential chemical analysis. Drilling followed by the injection of agarwood inducer is a common practice in Incense Tree farms, as it provides high‐grade agarwood (Chowdhury et al., 2016; Liu et al., 2013). However, this is the least frequent wound type in Hong Kong, possibly due to the complicated wound induction methods required.

The significant differences between the number of sawed wounds induced below and above breast height observed in this study were probably due to poor accessibility and preference. Sawed wounds are usually induced using a handsaw or chainsaw; therefore, it is more convenient and efficient to handle them below breast height. Inducing wounds near the trunk base is less noticeable than those above breast height, which may explain why more sawed wounds are induced below breast height than those above. In contrast, the number of trunk removals above breast height was higher than that below breast height. One possible explanation is that the poachers could revisit the tree to collect agarwood from the remaining part of the trunk. Many individuals in this study with trunks removed above breast height were still alive, and epicormic shoots and agarwood formation were observed on the wounds. Poachers might keep the tree alive by removing the trunk above breast height for the first collection and collecting newly formed agarwood again after a few months. Therefore, individuals with trunk removals below breast height may indicate that the poachers had already revisited the tree.

In addition, we found that some wounds were formed naturally on *A. sinensis*, which was mainly located at the axillary part between the two diverging trunks. This wound type is induced by mechanical forces due to the weight of the diverging trunks. As the wound was induced naturally, further analysis of more samples is required to examine whether any wound infection or agarwood formation occurred.

### 4.6 Future scope of photogrammetry applications for monitoring and conservation of *A. sinensis*

**FIGURE 4:**
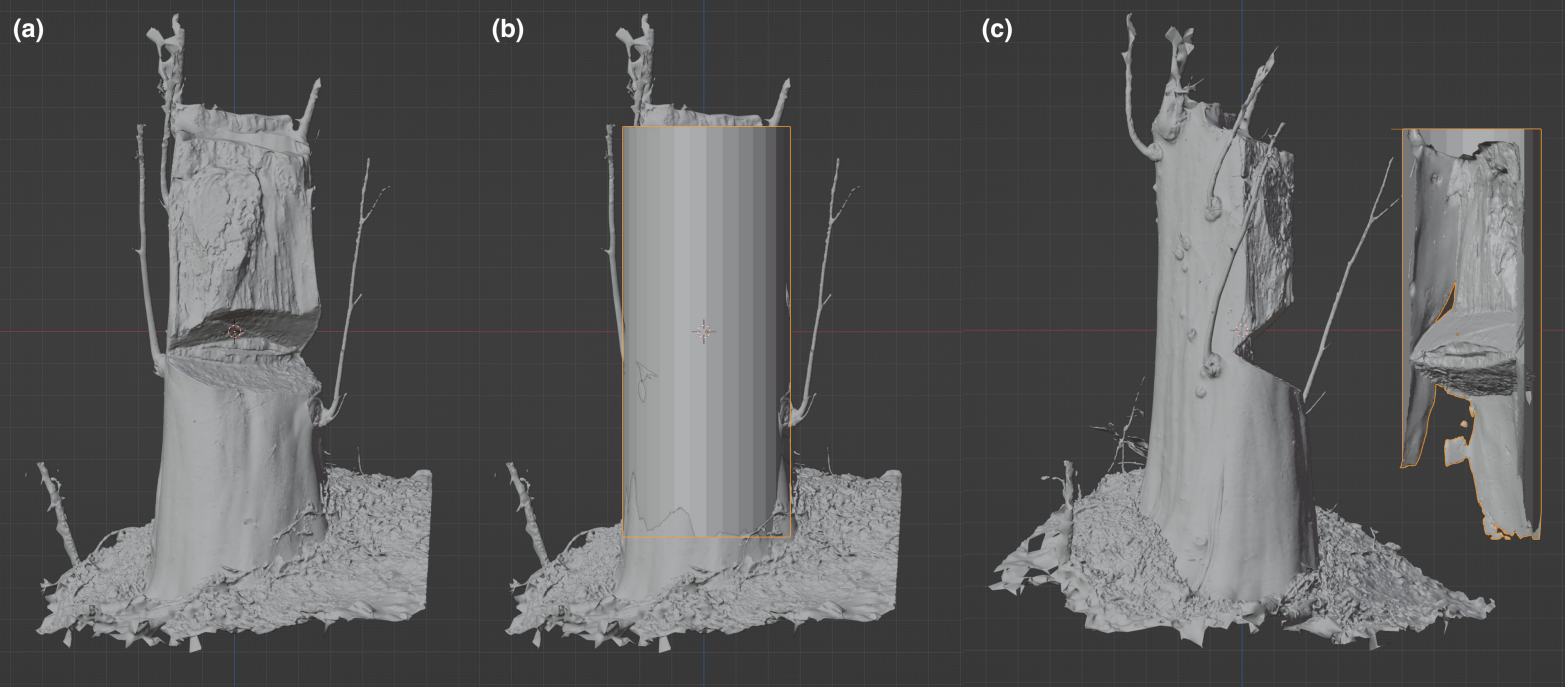
Recovery of wound shape and estimation of wound volume. (a) A 3D model of QH573. (b) A circular cylinder was added and scaled until it matched the shape of the remaining trunk as close as possible. (c) After the editing, the shape of the removed part of the trunk was recovered and the wound volume can be estimated.

Many studies have demonstrated the accuracy of photogrammetry in measuring tree diameter and volume (Bauwens et al., 2017; Mokroš et al., 2018, 2020; Morgenroth & Gómez, 2014; Surový et al., 2016). Hence, photogrammetry can potentially be used to measure wound volume. A 3D model, QH573, was used to demonstrate the potential applications of photogrammetry (Figure 4). The 3D model was imported into the editing software Blender, which had already been scaled to real size during editing. The original shape of the trunk was assumed to be cylindrical, as it was damaged prior to documentation, and the intact shape was unknown. A cylindrical mesh was added and scaled until it was as close as possible to the trunk surface. Subsequently, a Boolean modifier was applied by selecting “Difference” to combine both meshes in a subtractive way. The solver was selected as “Exact,” and “Self Intersection” was allowed. A mesh representing the removed part of the trunk was reconstructed, and the wound volume was estimated to be 43,000 cm^3^. The results demonstrated how to recover the shape of the removed wooden parts and estimate their volumes. If an intact individual is documented as a 3D model but later found to be damaged by poachers, the wound shape and volume can be accurately calculated by this method. This method could also provide a clear definition of incense tree wound types to be presented in court or in the legal statements of any related prosecution. Furthermore, this method may serve as scientific evidence to distinguish between natural and artificial wounds. The wound volume calculated using this method may serve as a reference to determine the extent of tree damage, which may facilitate legal proceedings.

Last but not least, the 3D photogrammetry can be used to document wound changes at different time points by periodically monitoring and reconstructing 3D models of the same wound. Therefore, this method will be useful for monitoring agarwood development, changes in wound size, decay rate, wound secretion, fungal formation, revisits by poachers, and the effects of wound dressing oil. Such results can provide good evidences to develop strategies of *A. sinensis* management and conservation.

## 5 FUTURE PERSPECTIVES

This study introduces an efficient 3D wound documentation of *A. sinensis* using photogrammetry to establish a new wound classification system. The results provide insights for local governments and non‐governmental organizations to facilitate the monitoring and conservation of *A. sinensis* in Hong Kong. In addition, the deliverables demonstrated a novel application of photogrammetry in terms of conservation. Following this study, some wounded individuals documented with research interests will be revisited regularly to monitor changes in wound situations. The wound documentation method can be further applied to other *Aquilaria* or similar species, including those that are threatened by illegal harvesting in other regions. For example, the endangered *Gyrinops* tree is also threatened by agarwood harvesting. In addition, photogrammetric documentation can be applied to other types of deep wounds caused by pathogens or insects. The 3D objective documentation can assist in tree condition and health assessments. When needed, all new wound‐related information can be presented as scientific evidence in ecological assessments and in any case of legal disputes. All in all, the new platform is multi‐directional and applicable in most aspects of work. Our vision for the future is that further research, education and practice of the 3D documentation platform are required to transfer the knowledge in long‐term conservation of incense tree and species diversity.

## AUTHOR CONTRIBUTIONS

**Ho Lam Wang:** Conceptualization (equal); data curation (lead); formal analysis (lead); investigation (lead); methodology (lead); project administration (equal); software (lead); validation (lead); visualization (lead); writing – original draft (lead); writing – review and editing (equal). **Tin Hang Wong:** Conceptualization (equal); data curation (equal); formal analysis (equal); investigation (equal); methodology (equal); project administration (supporting); software (equal); writing – original draft (supporting); writing – review and editing (supporting). **Ka Yip Eric Liu:** Conceptualization (equal); funding acquisition (equal); methodology (supporting); project administration (equal); resources (equal); supervision (equal); validation (equal); writing – original draft (equal); writing – review and editing (equal). **Ho Leung Ryan Tsang:** Conceptualization (supporting); data curation (equal); formal analysis (supporting); investigation (equal); methodology (equal); project administration (equal); validation (equal); visualization (equal); writing – original draft (equal); writing – review and editing (equal). **David Tai Wai Lau:** Conceptualization (lead); data curation (equal); formal analysis (equal); funding acquisition (equal); investigation (equal); methodology (equal); project administration (lead); resources (lead); supervision (lead); visualization (equal); writing – original draft (equal); writing – review and editing (lead).

## Data Availability

All the 3D models can be accessed on the “Virtual Carpological Herbarium” in the Shiu‐Ying Hu Herbarium (https://syhuherbarium.sls.cuhk.edu.hk/collections/3d‐digitized‐tag/wound/; Username: syhuherbarium; Password: @CUHK).
